# Antisynthetase Syndrome with Severe Interstitial Lung Disease in Pregnancy

**DOI:** 10.1155/2021/1150394

**Published:** 2021-07-26

**Authors:** Catalina I. Dumitrascu, David A. Olsen, Katherine W. Arendt, Carl H. Rose, Emily E. Sharpe

**Affiliations:** ^1^Department of Anesthesiology and Perioperative Medicine, Mayo Clinic, 5777 E Mayo Blvd, Phoenix, AZ 85054, USA; ^2^Department of Anesthesiology and Perioperative Medicine, Mayo Clinic, 200 1st St SW, Rochester, MN 55905, USA; ^3^Department of Obstetrics and Gynecology, Division of Maternal Fetal Medicine, Mayo Clinic, 200 1st St SW, Rochester, MN 55905, USA

## Abstract

Antisynthetase syndrome is a rare multisystem autoimmune disorder which clinically manifests with myositis, arthritis, interstitial lung disease, Raynaud phenomenon, and skin hyperkeratosis. Lung involvement represents the most severe form of disease and has rarely been reported in pregnancy. We present the case of a 22-year-old woman with antisynthetase syndrome and severe restrictive pulmonary disease who experienced a successful pregnancy and delivery. We discuss anesthetic considerations and highlight the importance of a multidisciplinary team approach in caring for parturients with multifactorial medical conditions.

## 1. Introduction

Antisynthetase syndrome is a severe autoimmune disorder characterized by the development of autoantibodies directed at aminoacyl-tRNA synthetase [1], a catalyzer enzyme important in the process of protein synthesis [2]. Clinical manifestations of antisynthetase syndrome include arthritis, myositis, Raynaud phenomenon, fever, skin hyperkeratosis, and interstitial lung disease (ILD) [2–4]. The onset of pulmonary symptoms is an indication of significant disease progression [1, 2], with a five-year mortality up to 45% [5]. Few cases of pregnancy in patients with antisynthetase syndrome have been previously reported [6–8], none of which described the anesthetic management. This report details the intrapartum anesthetic management of a patient with antisynthetase syndrome complicated by ILD. Written HIPAA authorization was obtained from the patient, and the EQUATOR guidelines were observed.

## 2. Case Presentation

A 22-year-old G3P0020 with a medical history significant for severe ILD, pulmonary hypertension, and exertional cyanosis secondary to antisynthetase syndrome was referred for antepartum obstetric anesthesia consultation. Antisynthetase syndrome was diagnosed three years prior based on a history of elevated creatinine kinase, inflammatory arthritis including polymyositis with myopathy demonstrated on electromyography, and anti-Jo-1 and anti-SSA antibodies. Associated features included fevers, mechanic's hands, and Raynaud's syndrome. Previous treatments included prednisone/methotrexate, prednisone/azathioprine, intravenous immunoglobulin therapy, and rituximab. Prednisone and rituximab were utilized during the pregnancy with improvement in rheumatologic but not respiratory symptomatology.

Our patient met with a multidisciplinary team comprised of cardiology, maternal-fetal medicine, rheumatology, and pulmonology for preconception counseling. Prepregnancy, she had developed substantial parenchymal restrictive disease with a forced vital capacity (FVC) 49% of predicted and diffusing capacity for carbon monoxide (DLCO) 34% of predicted. Furthermore, she had presumed pulmonary hypertension on echocardiography with estimated right ventricular systolic pressure of 39 mmHg. The patient did not require supplemental oxygen at rest or with mild activity prepartum. She was counseled regarding the anticipated significant maternal and fetal risks of pregnancy due to concern for her cardiopulmonary status. She elected to proceed with a pregnancy.

The patient underwent pulmonary rehabilitation twice weekly prepartum and throughout her pregnancy at an outside hospital in her home city. At our institution, pulmonary rehabilitation involves a multidisciplinary team of respiratory therapists, physical therapists, occupational therapists, social workers, and dieticians that educate patients on conservation of energy, reconditioning exercises, and breathing techniques. Pulmonary function testing at 13 weeks gestation revealed a FVC of 47% and DLCO of 40% of predicted. By 22 weeks gestation, her FVC had declined to 41% of predicted and DLCO was 31% of predicted. She was able to ambulate up to 2 mph with 1 liter per minute nasal cannula while maintaining a SpO_2_ of 90%, with the recommendation to utilize 3 liters per minute of oxygen with activity and at night. At 29 weeks gestation, she was evaluated for progressive dyspnea, with computed tomography angiography negative for pulmonary emboli. At 30 weeks gestation, FVC had increased to 48% of predicted, DLCO was 37% of predicted, and oxygen titration study again indicated 3 L per minute of oxygen was needed with activity.  Table 1 summarizes the patient's pulmonary function tests throughout her pregnancy. At 34 weeks gestation, she was admitted to the hospital due to oligohydramnios, considered to be secondary to chronic maternal hypoxemia. Following administration of antenatal corticosteroids, the decision was made to proceed with preterm delivery for oligohydramnios at 35 weeks gestation via cesarean delivery for fetal breech presentation.

Upon entry to the operating room, her SpO_2_ was 92% on room air, and supplemental oxygen was administered at 10 liters per minute via face mask. Following administration of 100 mg intravenous hydrocortisone, a spinal anesthetic was administered consisting of 1.6 ml 0.75% bupivacaine with dextrose 8.25%, 15 mcg fentanyl, and 150 mcg morphine. No intravenous sedative medications were administered. A T4 spinal level was achieved, and an uncomplicated primary low transverse cesarean was performed with delivery of a female infant weighing 2560 grams with Apgar scores of 8 and 9 at 1 and 5 minutes, respectively. Following delivery, supplemental oxygen was discontinued and SpO_2_ remained ≥96%. Her initial postpartum recovery was uneventful, and she was discharged to home on postoperative day 4. On postoperative day 5, she returned for evaluation of new-onset fever ultimately ascribed to an antisynthetase flare.

## 3. Discussion

ILD in antisynthetase syndrome presents with a restrictive spirometry pattern, with FVC and DLCO the best predictors of disease extent [1, 3]. The most common antibody in antisynthetase syndrome has been shown to be anti-Jo-1 [4, 5], while anti-PL7 and anti-PL12 are involved in the most severe forms of ILD [1, 4]. Concomitant anti-SSA antibodies may also be associated with more extensive pulmonary disease [4, 9].

Given its rarity, few cases of antisynthetase syndrome have been reported in pregnancy [6–8, 10–12]. In 1994, a case report of a 24-year-old woman who developed acute polymyositis and autoantibodies to PL7 in the second trimester with subsequent fetal loss was published, though she did not develop pulmonary disease [12]. Green et al. described a patient with a preconceptual diagnosis of antisynthetase syndrome who completed a successful pregnancy, highlighting the role of a multidisciplinary team for an optimal outcome [6]. A case of antisynthetase syndrome with severe pulmonary involvement was described, in which a healthy neonate was delivered via cesarean [10]. Postpartum onset of antisynthetase syndrome was reported in a patient characterized by the development of Henoch–Schönlein purpura and nephritis [11]. Lastly, Vancsa et al. assessed the prevalence and outcomes of idiopathic inflammatory myopathies in pregnant patients, only two of which were diagnosed with antisynthetase syndrome [8]. They reported that 14% of women affected with this group of diseases were of reproductive age, with the increased risk of peripartum complications in the setting of active signs of disease [8].

A multidisciplinary team including maternal-fetal medicine, pulmonology, rheumatology, cardiology, anesthesiology, and neonatology physicians is essential in the care of a patient with antisynthetase syndrome. Counseling should begin prior to conception, given that outcomes are improved when conception occurs during a period of disease remission with an optimized medication regimen [6]. Treatment typically includes corticosteroids at the lowest disease-modifying dose, with the possible addition of immunosuppressive agents [6]. Monitoring of the fetus with serial growth scans is advised, and early screening for gestational diabetes mellitus in the setting of maintenance corticosteroid is also suggested [6].

Anticipating physiologic changes of pregnancy and their effects on a patient with restrictive lung disease is important and may necessitate regular repeat cardiopulmonary tests. There was a significant concern regarding our patient's ability to compensate, as she had limited exercise capacity, increased hypoxia on exertion, pulmonary hypertension, and marked restrictive lung disease. With a growing abdomen and elevation of the diaphragm, respiratory reserve is further decreased, leading to worsening of restrictive physiology. Our patient underwent three spirometry evaluations during her pregnancy, which were remarkably stable (FVC 47%, 41%, and 48% and DLCO 40%, 31%, and 37% of predicted). Oxygen requirements and titration studies remained stable throughout her pregnancy, though they were increased from prepregnancy requirements. Prepregnancy, the patient did not require supplemental oxygen at rest, with activity, or overnight. Throughout her pregnancy, she required supplemental oxygen with activity and overnight. Interestingly, this stability matches prior reports, as Lapinsky et al. note that though rare, restrictive lung disease in pregnancy is often tolerated well and cesarean delivery is typically reserved for obstetric indications [13]. We speculate that this stability may be attributed to consistent pulmonary rehabilitation, prepartum multidisciplinary evaluation, optimized medication management prior to conception, and her unique disease process. Cesarean delivery in our patient was performed due to obstetric indications (breech presentation) rather than ILD.

In a spontaneous vaginal delivery, consideration of an early epidural for labor analgesia may assist in blunting the pulmonary and cardiovascular changes of painful labor. In the setting of a cesarean, we recommend a neuraxial technique such as spinal anesthesia, epidural, or combined spinal-epidural. All of the typical risks associated with a general anesthetic in pregnancy apply including increased difficult intubation, aspiration, decreased uterine tone, risk of lung injury with mechanical ventilation, and decrease in maternal satisfaction. There is also a significant risk of postoperative pulmonary complications in patients with ILD undergoing general anesthesia, including acute lung injury, pneumonia due to aspiration or microaspiration, atelectasis, pneumothorax, pulmonary embolism, and ventilatory-induced lung injury in the setting of reduced lung compliance leading to an increase in morbidity and mortality [14]. Neuraxial anesthesia provides the ability to maintain spontaneous respirations to avoid hypoxia and hypercapnia that can lead to worsening pulmonary hypertension. Furthermore, it allows for the provision of postoperative analgesia with intrathecal opioids.

A concern regarding neuraxial anesthesia in ILD includes paralysis of accessory muscles of respiration with midthoracic or higher levels of anesthesia. An epidural or low-dose sequential combined spinal epidural could be considered for incremental titration of the onset of anesthesia. Though epidural anesthesia can reduce vital capacity and forced expiratory volume in one second, the effect is minimal when local anesthetic travels from the lumbar to midthoracic regions [15]. In the setting of significant reduction of lung function, such as that of a high thoracic or low cervical block, the overall effect is nonetheless a reduction of postoperative pulmonary complications [15].

The administration of stress-dose steroids should be considered to decrease the risk of adrenal insufficiency in patients taking high-dose corticosteroids [6]. Intravenous sedation and pain medications such as fentanyl should be judiciously used given the risk of hypoventilation, decrease pulmonary reserve, and worsening pulmonary hypertension. Lastly, in the event of a postpartum hemorrhage, oxytocin should be used as a first line uterotonic, with misoprostol as secondary. Carboprost should be judiciously used as it may cause bronchoconstriction and worsen pulmonary hypertension. Methergonovine can also worsen pulmonary hypertension and therefore should also be avoided if possible.

Parturients with complex rheumatologic and pulmonary disease benefit from a multidisciplinary team, preconception planning, and close monitoring throughout pregnancy. Patients with severe restrictive pulmonary disease can undergo successful pregnancy and delivery and likely benefit from neuraxial anesthesia or analgesia. In patients with severe pulmonary disease, fear of hypoxic respiratory failure during cesarean delivery should not preclude a neuraxial anesthetic.

## Figures and Tables

**Table 1:** Pulmonary function tests.

	Prepregnancy	13 weeks	22 weeks	30 weeks	9 months postpregnancy
TLC	56%	54%	53%	56%	59%
FVC	49%	47%	41%	48%	49%
FEV1	49%	51%	45%	53%	52%
FEV1/FVC	99%	108%	109%	110%	104%
DLCO	34%	40%	31%	37%	35%
O_2_ requirements with activity and at night	None	3L NC	3L NC	3L NC	None

Values are represented as fractions of predicted normal values. Normal values are the following (95% confidence interval): TLC, total lung capacity (80%–120%); FVC, forced vital capacity (80%–120%); FEV1, forced expiratory volume at one second (80%–120%); FEV1/FVC, percentage of the FVC expired in one second (within 5% of the predicted ratio); DLCO, diffusing capacity of the lung for carbon monoxide (>60% to <120%).

## Data Availability

The data that support the conclusions of this case report are restricted by HIPPA in order to protect patient privacy. Data are available upon request from the corresponding author, CID, for researchers who meet the criteria for access to confidential data.
